# Success Rates of Assisted Reproduction in Couples with Poor Ovarian Response and Oligospermia

**DOI:** 10.3390/cells14191492

**Published:** 2025-09-24

**Authors:** Jakub Wyroba, Joanna Kochan, Maciej Brązert, Paweł Kordowitzki

**Affiliations:** 1 Malopolski Institute of Fertility Diagnostics and Treatment—KrakOvi, 30-118 Krakow, Poland; jakub.wyroba@krakovi.med.pl (J.W.); joanna.kochan@urk.edu.pl (J.K.); 2 Fertility Disorders Clinic, Andrzej Frycz Modrzewski Krakow University, 30-705 Krakow, Poland; 3 Department of Animal Reproduction, Anatomy and Genomics, University of Agriculture in Kraków, 30-705 Krakow, Poland; 4 Department of Diagnostics and Treatment of Infertility, Poznan University of Medical Sciences, 61-701 Poznan, Poland; maciejbrazert@ump.edu.pl; 5 Department of Basic and Preclinical Sciences, Faculty of Biological and Veterinary Sciences, Nicolaus Copernicus University, 87-100 Torun, Poland; 6 Department for Gynaecology, Charité Medical University, 10117 Berlin, Germany

**Keywords:** poor ovarian response, in vitro fertilization, oligospermia, women, men

## Abstract

Recent progress in assisted reproductive medicine has introduced novel therapeutic possibilities for couples experiencing various reproductive challenges or subfertility. A critical concern in this field is the diminished ovarian response to hormonal treatments preceding ovum pickup, necessitating personalised and optimised protocols to enhance ovarian response across different age groups. Furthermore, a common male factor in IVF couples, oligozoospermia, characterised by a low sperm count, significantly impacts the success rates of assisted reproductive technologies, posing an increasing challenge for in vitro fertilisation clinics. Lifestyle choices, dietary habits, and overall health behaviours have also demonstrably affected fertility outcomes in the 21st century. This original article aims to highlight the synergistic importance of both partners’ health, specifically addressing poor ovarian response and oligozoospermia, in achieving successful conception. Our study analysed intracytoplasmic sperm injection outcomes in couples affected by both aforementioned conditions and proposed an optimal management strategy. This study shows that oligozoospermia significantly reduced ICSI fertilisation and cleavage rates. Poor ovarian responders experienced more cancelled cycles due to fewer embryos. While blastocyst rates relative to zygotes were comparable, overall success was lower in groups with male factor infertility and poor ovarian response, necessitating personalised treatment approaches.

## 1. Introduction

One of the main factors determining the success of in vitro fertilisation (IVF) or intra-cytoplasmic sperm injection (ICSI) in human reproductive medicine is the ovarian response to hormonal stimulation and obtaining a sufficient number of oocytes, allowing the development of euploid blastocysts, subsequent pregnancy, and birth of healthy offspring [1]. It is assumed that the optimal number of oocytes after ovarian stimulation is 10–15 [2]. One of the main female factors in deciding to undertake the IVF procedure is low ovarian reserve and, consequently, poor ovarian response (POR) to stimulation and a small number of collected oocytes [3]. POR signifies an inadequate follicular output and retrieval of limited oocytes after controlled ovarian stimulation, significantly reducing treatment success. Often linked to advanced maternal age and diminished ovarian reserve, POR can also appear in younger patients. Achieving an optimal oocyte yield (typically 10–15) is vital for euploid blastocyst development and pregnancy rates.

Thus, personalised stimulation protocols are essential for effective POR management and improved IVF outcomes. Recognising and effectively addressing POR through individualised stimulation protocols and precise diagnostic criteria is paramount to optimising treatment success and improving patient outcomes in assisted reproductive technologies [4,5,6]. According to various reports, POR occurs in about 30% of all ART cycles in women [5,6]. Based on a multicentre cohort study, POR patients have a 50% lower cumulative delivery rate per IVF/ICSI cycle than normal responders [7]. Other studies show that pregnancy rates are 10% and live birth rates are 7% per cycle in POR patients younger than 40, and they decrease after the age of 40 years [8,9]. Moreover, POR patients are at high risk of cycle cancellation due to the lack of oocytes or embryos, which causes the couple enormous stress and can lead to their reluctance to undergo further IVF cycles. Therefore, POR patients are a group that requires a lot of care, but it is also a challenge for doctors to choose optimal strategies to manage the treatment of these patients. Additionally, the management of ART procedures in POR patients becomes complicated when the male factor also appears. The male factor is responsible for about 30% of infertility cases and is a reason for using ART [1,10].

The male factors predisposing to the use of ART include abnormalities such as oligozoospermia (concentration < 16 million/mL), asthenozoospermia (sperm motility < 42%), and teratozoospermia (morphologically normal sperm < 4%) (World Health Organisation/WHO guidelines, 2021). Most often, these abnormalities occur together. Oligozoospermia is defined as a severe reduction in sperm concentration, and it is frequently associated with male infertility. The diagnosis of oligozoospermia is made based on a sperm concentration lower than 16 million sperm per millilitre of ejaculate, based on at least two standard sperm analyses performed [11]. Notably, oligozoospermia is most strongly associated with an increased frequency of chromosomal abnormalities in sperm, which leads to a reduced fertilisation rate and blastocyst rate [12,13]. Moreover, it has been proven that there is an increased risk of de novo chromosome abnormalities in offspring after intracytoplasmic sperm injection using sperm from men with oligozoospermia [14,15]. Due to the fact that the frequency of POR is approximately 30% and that of the male factor is also approximately 30%, there is a high probability of the coexistence of both POR and oligozoospermia in one couple undergoing the IVF/ICSI procedure.

We hypothesise that oligozoospermia may significantly reduce fertilisation and cleavage rates and that poor ovarian responders face higher cycle cancellation rates. Therefore, the aim of our study is to analyse ICSI outcomes in couples with POR and oligozoospermia and try to determine the optimal treatment management for these couples as a potential personalised strategy for IVF clinics.

## 2. Materials and Methods

This was a retrospective study of 817 cycles from 750 couples (first and second cycles) who underwent an intracytoplasmic sperm injection procedure at the Kraków Clinic in Kraków (Poland) from 2021 to 2024.

### 2.1. Study Design and Inclusion Criteria

**Figure 1:**
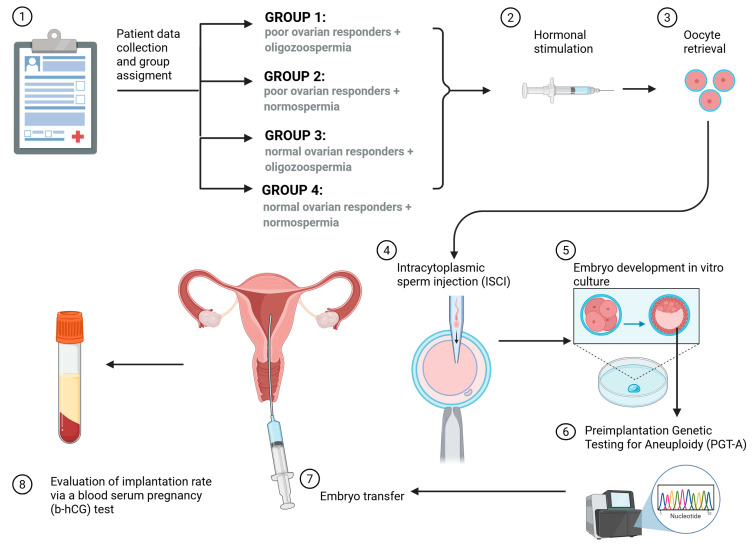
Scheme showing the study design and inclusion criteria. Here, ICSI outcomes across four patient groups (I–IV) were investigated, categorised by ovarian response and semen quality. Poor ovarian response was defined by an Anti-Müllerian hormone level below 1.2 ng/mL and retrieval of four or fewer oocytes, while oligozoospermia was identified according to the 2021 WHO criteria as a sperm concentration below 16 million/mL. To mitigate the confounding effect of advanced maternal age, all participating women were 38 years of age or younger. In cycles with PGT-A, blastocysts were biopsied at day 5, using the same micromanipulator and microscope used for ICSI.

The study (Figure 1) analysed the ICSI results of four groups of couples depending on the ovarian response (PORs (poor ovarian responders) and normal ovarian responders) and semen quality (oligozoospermia, normozoospermia). In Group IV (normal ovarian responders + normozoospermia), indications for ICSI included other female factors such as blocked fallopian tubes or low-grade endometriosis. In our study, POR was defined as AMH < 1.2 ng and ≤4 retrieved oocytes. Oligozoospermia was defined according to the 2021 WHO guidelines as sperm concentration < 16 million/mL. Women were aged ≤38 years to eliminate the influence of advanced maternal age.

### 2.2. Clinical Protocols

Patients were treated using either the long agonist protocol or the short antagonist protocol. The type of protocol used depended on the AMH level and the overall hyperstimulation risk.

#### 2.2.1. Long Agonist Protocol

To initiate the treatment protocol, patients began receiving the GnRH agonist, triptorelin (Decapeptyl, 1 mg/d, sc), one week prior to their anticipated menstrual period (cycle day 18–23). Following confirmation of successful pituitary downregulation, indicated by blood serum oestradiol (E2) levels below 40 pg/mL, ovarian stimulation was initiated. This stimulation involved a consistent daily dosage of 150–300 IU of recombinant follitropin alfa (rFSH, GonalF, sc), with the potential addition of 75–150 IU of menotropin (hMG, Menopur).

#### 2.2.2. Antagonist Protocol

Either Cetrotide (Cetrorelix, 0.25 mg/d, sc) or Ganirelix (0.25 mg/d), both GnRH antagonists, were given starting when the largest follicle reached a diameter of 14 mm. From days 2–4 of the cycle, rFSH/hMG was started. Until the day of human chorionic gonadotropin (hCG) treatment, which is when the leading follicle reached a diameter of 18 mm or more and at least three follicles reached a diameter of 17 mm or more, the agonist and antagonist protocols were followed. At that point, the rFSH was discontinued. Additionally, 36 h prior to the intended day of oocyte retrieval, a single subcutaneous dose of 10,000 IU of hCG (Eutrig) or 6500 IU of rhCG (Ovitrelle) was given. In an antagonist cycle, a single 2 mg sc bolus of triptorelin was used as the trigger, and a freeze-all strategy was implemented if there was a chance of OHSS. All follicles measuring 12 mm or more were aspirated. After that, the oocytes were fertilised using ICSI, and three to five days later, just one embryo was moved. The quantity and grade of the embryos determined the transfer selection on days 3 or 5. ET was selected on day 3 if there was only one embryo or if the embryos were of poor quality. Intravaginal luteal support in the form of progesterone was used. From the day following oocyte retrieval until a serum pregnancy (b-hCG) test was conducted 17 days later, progesterone (Cyclogest, 400 mg twice daily) was administered. Implantation rate was calculated upon a positive b-hCG test. Clinical pregnancy was defined by the ultrasound confirmation of an intrauterine gestational sac after eight weeks of gestation with visible foetal cardiac activity.

### 2.3. Ovarian Stimulation Monitoring in ICSI

All patients underwent baseline blood sampling and transvaginal sonography (TVS) on day 2 or 3 of the pretreatment cycle. Blood samples for hormone analysis were taken on cycle days 2–3 (oestradiol, FSH, LH) and 5–6 (oestradiol) for TVS monitoring of response throughout the treatment cycle, on the day of hCG administration (oestradiol, progesterone), and on days 8–9 (oestradiol). As clinically indicated, additional TV surveillance was conducted.

### 2.4. Preparation for Frozen Embryo Transfer

On the first, second, or third day of the cycle, oral oestradiol therapy was initiated to prepare the endometrium and prevent the spontaneous development of follicles. Oral oestradiol was given gradually: 2 mg/day on days 1–7, 4 mg/day on days 8–12, and 6 mg/day on the days leading up to embryo transfer. To measure endometrial thickness and rule out the presence of a dominant follicle, a vaginal ultrasound was typically performed 12 to 14 days after beginning E2 administration. Progesterone (P4) supplementation (Cyclogest, 400 mg twice daily) was started when the endometrial thickness exceeded 7 mm, and the timing of FET was planned accordingly. To rule out any cyst or corpus luteum remaining from the prior cycle, TVS was conducted on days 2 or 3 of menstruation for t-NC. The majority of cycle cancellations occurred on day 2 or 3 of menstruation at a serum P4 level of more than 1.5 ng/mL. Typically, endocrine monitoring, which includes measuring serum E2, LH, and P4, began when the dominant follicle reached day 8–10, along with transvaginal ultrasonographic monitoring. The day of ovulation was precisely recorded after regular endocrine and ultrasonographic monitoring, either daily or on alternate days, with an average diameter of roughly 15 mm, to plan the timing of FET.

### 2.5. Laboratory Protocols

After 2–5 days of sexual abstinence, semen samples were collected by masturbation and analysed using the SCA^®^ CASA system (Microptic S.L.U., Barcelona, Spain) according to the WHO guidelines (WHO guidelines 2021) in order to qualify the patient for the in vitro fertilisation procedure using the ICSI method. The following parameters were evaluated: motility and concentration, morphology, vitality, and DNA fragmentation. On the day of the partner’s ovarian puncture, the semen was collected and evaluated again. A sperm sample was prepared for ICSI using the density gradient method (Gynemed, Sierksdorf, Germany).

ICSI was performed using a Nikon Eclipse CS100 microscope and an RI Integra 3 micromanipulator (Research Instruments, Dortmund, Germany), following the standard technique. Embryos were in vitro cultured in SAGE^®^ medium (Origio, Ballerup, Denmark) in an atmosphere of 6.0% CO_2_, 5.0% O_2_, and balanced N_2_ at 37 °C. The extent of expansion was used to grade blastocysts in accordance with the Gardner scoring system. Blastocysts were biopsied on day 5 during cycles that included PGT-A, using the same microscope and micromanipulator that were used for ICSI. A Vitrolife Octax^®^ laser (Vitrolife, Kungsbacka, Sweden) was used to puncture the zona pellucida for 250 microseconds. For PGS referral to Igenomix Inc. in Valencia, Spain, the biopsied TE cells were washed with D-PBS and then placed in 0.2 mL polymerase chain reaction (PCR) tubes and subjected to next-generation sequencing (NGS) analysis. The blastocysts were vitrified after being incubated for 1.5 h in Sage^®^ medium following the biopsy. Post-biopsy blastocysts and blastocysts without PGT-A were vitrified following the manufacturer’s instructions, using the Cryotop device (Kitazato, Shizuoka, Japan) and the Kitazato^®^ media. Before transfer, blastocysts were maintained in Kitazato media for at least 1.5 h, and then they were placed in Sage^®^ medium or EmbryoGlue^®^ medium (Vitrolife, Kungsbacka, Sweden). In EmbryoGlue^®^ media, more than 95% of fresh ETs and FETs were carried out.

### 2.6. Statistical Analysis

The chi-square test was used to examine non-parametric data, such as variations in the percentage values between groups, because the measured variables did not adhere to a particular distribution. No assumptions were made about nominal and ordinal scales. Two-way ANOVA was used to compare parametric data, which were presented as means ± SD. When the *p*-value was less than or equal to 0.05, differences were deemed significant. The R software, version 4.4.1 (R Project for Statistical Computing), and PQStat 1.6.2 (PQStat Soft, Poznan, Poland) were used to conduct the statistical analysis. The paired t-test and Fisher’s exact test were used to evaluate variations between continuous and categorical variables, respectively.

## 3. Results

The study involved an analysis of 817 ICSI cycles (Table 1). In the groups with poor ovarian response, 402 (Group I) and 440 (Group II) oocytes in metaphase II (MII) were used for ICSI, with an average of 2.01 and 2.2 oocytes per patient, respectively. Conversely, in the groups with a normal ovarian response, significantly more oocytes were fertilised, 1620 (Group III) and 1560 (Group IV), on average 8.1 and 7.8 per patient, respectively. In both groups (I and III) with oligozoospermia, we detected a reduced fertilisation rate regardless of ovarian response in comparison to the group with normospermia (Group I: 54% and Group III: 60% vs. Group II: 71% and Group IV: 85%, *p* < 0.05).

A decrease in cleavage rate was observed in the oligozoospermia groups. However, the blastocyst rate in relation to the number of zygotes was similar and was as follows: Group I: 38%; Group II: 42%; Group III: 44%; Group IV: 45% (*p* > 0.05). The percentage of cancelled cycles due to a lack of embryos for ET was the lowest in the groups with normal ovarian response (Group III 9%, Group IV 5%). In groups with poor ovarian response, there were significantly (*p* < 0.05) more cancelled cycles than in the normal responder groups (GI 30%, GII 24%).

**Figure 2:**
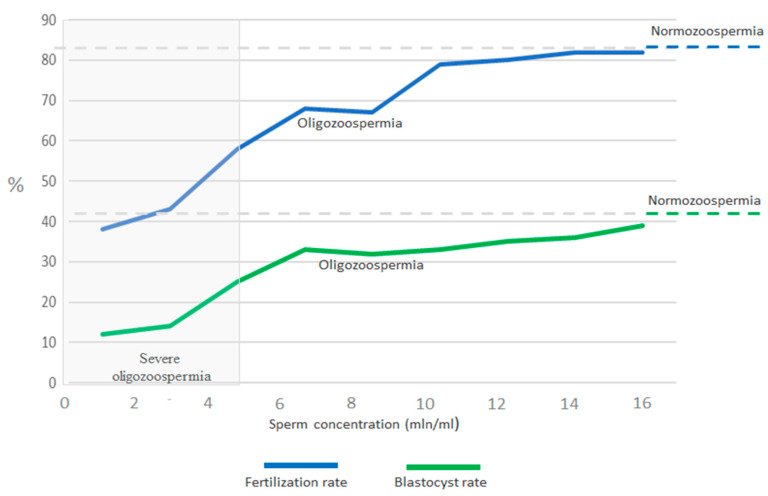
Fertilisation rate (blue line) and blastocyst rate (green line) in patients with oligospermia and normospermia. The y-axis shows the respective rates in %, and the x-axis shows the sperm concentration in millions per ml, and severe oligozoospermia is shown on the very left.

**Figure 3:**
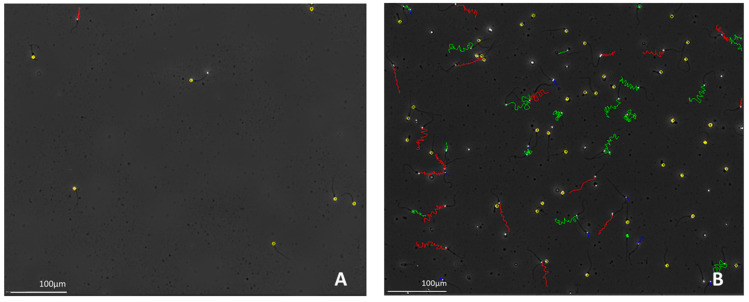
Representative images from analysis of motility and concentration using the SCA^®^ system: (**A**) severe oligozoospermia, (**B**) normozoospermia.

Interestingly, in the groups with oligozoospermia (Groups I and II), severe oligozoospermia (<5 million/mL) was at a similar level (35 vs. 37%). Based on Figure 2, at a concentration of 1 million/mL, the fertilisation rate is twice as low as in the normozoospermia counterparts, and also the sperm motility differs (Figure 3). At concentrations >10 million/mL, the fertilisation rate remains at a level similar to that in groups with normozoospermia. The blastocyst rate at a concentration of 1 million/mL is only 10%, and at >7 million/mL, it stabilises at the level of 30–35%, compared to 41% in normozoospermia.

In the POR groups (I, II), due to a reduced number of blastocysts or their absence, significantly fewer (*p* < 0.05) couples choose preimplantation genetic testing of embryos (PGT-A), compared to couples from Groups III and IV (Group I: 25%; Group II: 29% vs. Group III: 43%; Group IV: 49%). Despite the differences in the number of PGT-A tests performed, no differences in the euploid rate were detected between the groups (Group I: 51%; Group II: 52%; Group III: 49%; Group IV: 52%)

**Figure 4:**
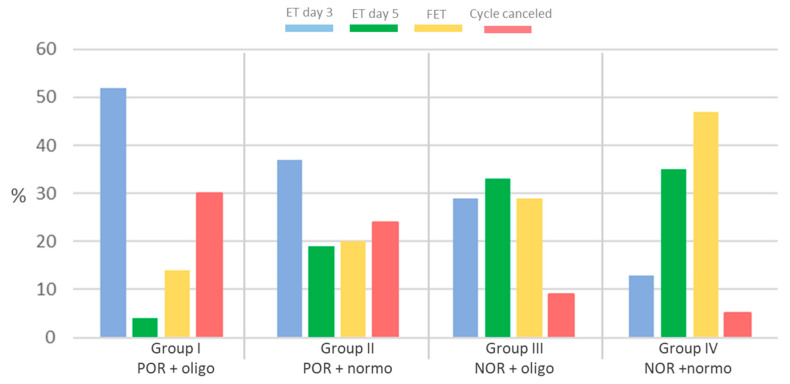
Graph showing the rates of conducted embryo transfers (ETs) and cancelled cycle rates in the four patient groups.

Figure 4 shows the ET strategies for the analysed groups. In Group I, the highest number of embryo transfers was recorded on day 3 (52%) and the lowest number on day 5 (4%), as well as on the day of FET (14%), when compared to the other groups. In Group IV, the highest number of FETs (47%) and ETs was recorded on day 5 (35%) compared to the other groups. Along with the highest percentage of ETs on day 3 in Group I, the lowest clinical pregnancy rate was also recorded in the same group (16%), and the highest pregnancy rate was recorded in Group IV (56%), as presented in Table 1.

**Figure 5:**
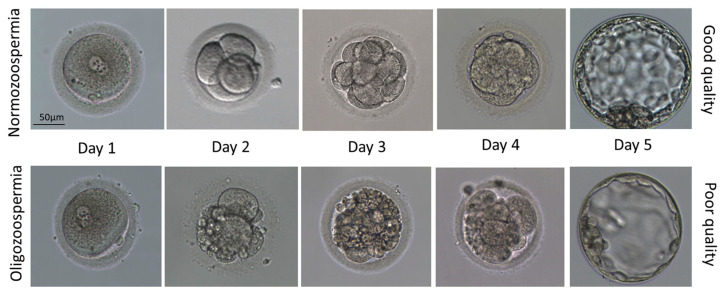
Representative images of good- and poor-quality embryos depending on the quality of semen (normozoospermia, oligozoospermia) on day 1 (zygote), day 2 (4-cell stage), day 3 (8–16 cell stage), day 4 (morula stage), and day 5 (blastocyst stage).

**Figure 6:**
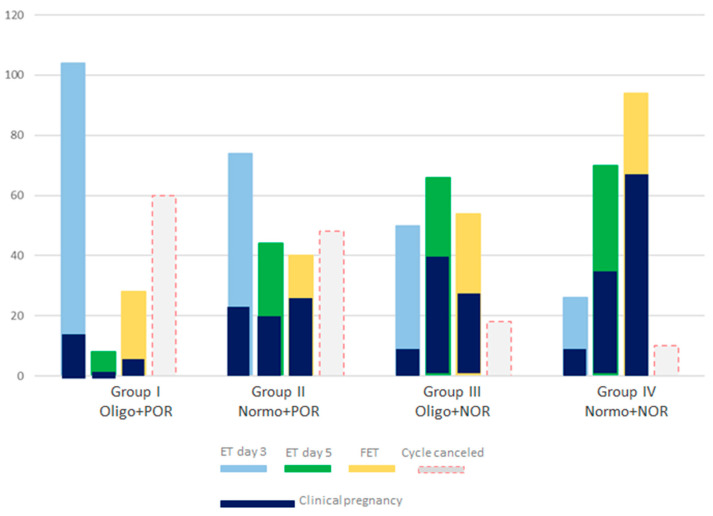
Total clinical pregnancy rate of the four patient groups analysed. The y-axis shows the total number of respective implanted embryos for day 3 (light blue bar) embryo transfer (ET), for day 5 (green bar) ET, and for the frozen/thawed (yellow bar) embryo transfer (FET). The bar with the dashed red line shows the total number of cancelled cycles in couples in the four analysed groups.

The morphological differences between the day 3 and day 5 embryos are shown in the representative microscopic pictures in Figure 5. The clinical pregnancy rate (Figure 6) was the highest for the FET procedure in each group.

## 4. Discussion

The introduction of in vitro fertilisation in 1978 has led to numerous advancements in hormonal treatment protocols designed to enhance ovarian stimulation outcomes and improve the number of mature oocytes retrieved during ovum pick-up procedures. Intracytoplasmic sperm injection, a more sophisticated reproductive technology developed in the 1990s, is utilised when standard IVF is not feasible due to sperm pathologies or motility issues. ICSI involves the direct injection of a single sperm into an egg, thereby addressing challenges related to low sperm count or poor motility. The application of ICSI has expanded considerably in recent years, now constituting roughly two-thirds of all fresh assisted reproductive technology treatments conducted worldwide [16]. As shown in this study, the fertilisation and cleavage rates were lower in the oligospermic groups compared to the normospermic counterparts (Table 1), even though the ICSI method was used. It is noteworthy that some clinics have started to rely almost solely on ICSI for every fresh cycle, raising questions about its application for patients who do not have male fertility issues [1,17]. Research in the United States shows that more ICSI procedures are being performed even when male infertility is not a factor, indicating this trend is more common among couples with no male issues [18]. As presented in Table 1 and Figure 6, the clinical pregnancy rate was the highest in the good ovarian responder group with normospermic partners after performing ICSI and ET. However, older studies have not proven that ICSI yields better live birth rates compared to IVF for these couples [19,20]. In regard to our data related to men with oligospermia, the cycle cancellation rate, meaning that the couple did not finish the entire ART procedure properly, was significantly higher for couples with poor ovarian response and oligospermia (Table 1). Noteworthy, oligozoospermia is a common parameter of semen analysis in infertility clinics, with increasing importance during the last decades [21,22]. As presented in Figure 2, severe oligozoospermia, meaning a condition when the sperm concentration is less than 5 × 10^6^ sperm/mL, has a significantly negative impact on ICSI outcomes, as can be seen in the low fertilisation rate (Figure 6). Our data clearly shows that both male and female infertility remains a major clinical concern, affecting approximately 10–15% of reproductive-aged couples. In this retrospective study, two particularly challenging conditions encountered in assisted reproductive technologies have been discussed, namely oligospermia and poor ovarian response. When these conditions co-occur within a couple, they substantially lower the chances of successful fertilisation, embryo development, and pregnancy (Table 1), requiring specialised clinical management.

Clinicians should keep in mind that oligospermia can arise from diverse etiologies, including genetic defects, hormonal dysfunction, varicocele, systemic illness, and environmental exposures, although idiopathic cases are common. Poor ovarian response, defined by criteria such as the Bologna and POSEIDON classifications, is most often observed in women with diminished ovarian reserve or advanced reproductive age, as has recently been shown [23]. These patients typically yield a reduced number of oocytes even with optimised stimulation protocols (please note the Materials and Methods section), limiting the number of embryos available for transfer or cryopreservation. Various stimulation strategies—ranging from high-dose gonadotropins to mild stimulation protocols, the use of adjuvants such as androgens, growth hormone supplementation, and dual stimulation cycles—have been proposed to improve outcomes, though success remains variable. Clinically, the coexistence of oligospermia and POR necessitates a highly individualised and multidisciplinary approach. Careful sperm selection, potentially using advanced sorting methods or DNA fragmentation testing, coupled with tailored ovarian stimulation regimens, is crucial. Early consideration of embryo banking, utilisation of blastocyst-stage transfers, and the possibility of using donor gametes should be part of comprehensive counselling offered to these couples. The emotional burden associated with dual-factor infertility is significant. As such, psychological support services should be routinely integrated into the management plan. Close monitoring, transparent communication regarding prognosis, and setting realistic expectations are essential to minimise patient distress and optimise adherence to treatment.

## 5. Conclusions

This article advocates for a personalised approach to managing couples facing oligozoospermia and poor ovarian response. Our analysis of 817 ICSI cycles highlights critical challenges for couples with oligozoospermia and poor ovarian response. Oligozoospermia significantly reduced fertilisation and cleavage rates irrespective of ovarian response, with sperm concentration directly correlating with these outcomes. While blastocyst rates relative to zygotes were comparable, poor ovarian responders faced higher cycle cancellation rates due to fewer embryos. Despite varied PGT-A usage, euploid rates remained consistent across groups. These findings underscore the complexity of managing such cases, emphasising the need for patient-centric strategies to mitigate the impact of male factor infertility and optimise ART success, especially in the context of diminished ovarian reserve. While donor sperm can enhance conception chances, many men prefer treatments for biological offspring. For male factor infertility, in vitro fertilisation or intracytoplasmic sperm injection utilising sperm from a testicular biopsy are common interventions. Research indicates a correlation between elevated serum follicle-stimulating hormone and impaired spermatogenesis, as well as a notable impact of low serum inhibin B on male fertility. Consequently, these hormonal markers should inform individualised treatment strategies. Successfully managing couples with both oligospermia and poor ovarian response presents a significant challenge in assisted reproductive technology. We suggest a tailored treatment plan, integrating advanced laboratory methods, customised stimulation protocols, and robust psychosocial support, which offers the optimal pathway to pregnancy for this patient group. Emerging therapeutic avenues, including stem cell and mitochondrial therapies, may expand future treatment possibilities. Further investigation is warranted to fully understand these aspects, particularly given the global increase in fertility issues affecting both genders.

## Figures and Tables

**Table 1:** Results of the different groups tested. POR—poor ovarian responder; NOR—normal ovarian responder; AMH—Anti-Mullerian hormone; BMI—body mass index; calculated as weight in kilograms divided by the square of the height in metres; a:b, b:c, c:d—values with different superscripts within the same column differ significantly (*p* < 0.05); a:c, a:d—values with different superscripts within the same column differ highly significantly (*p* < 0.001).

		Group I POR + Oligozoospermia	Group II POR + Normozoospermia	Group III NOR + Oligozoospermia	Group IV NOR + Normozoospermia
**No of couples (n)**		188	184	180	198
**N of cycles (n)** **No of cycles/couple** **mean ± SD,**		208 1.1 ± 0.2	202 1.09 ± 0.1	197 1.09 ± 0.2	210 1.06 ± 0.2
**Female age, y,** **mean ± SD,**		35.5 ± 0.2	35.2 ± 2	34.3 ± 3	34.5 ± 2
**Female BMI,** **mean ± SD**		22.6 ± 3	23.3 ± 4	22.9 ± 3	23.1 ± 3
**AMH ng/mL** **mean ± SD**		0.8 ± 0.5	0.7 ± 0.5	3.4 ± 2	3.8 ± 3
**Male age, y,** **mean ± SD**		37 ± 2	36 ± 3	36 ± 3	35 ± 4
**Male BMI,** **mean ± SD**		24 ± 3	23.7 ± 4	23,9 ± 3	24,1 ± 2
**Sperm concentration** **mln/mL, mean ± SD,**		7 ± 3 ^a^	48 ± 11 ^c^	7 ± 5 ^a^	49 ± 14 ^c^
**Ovarian** **stimulation protocol**	Antagonist, n (%)	175/208 84% ^a^	174/202 86% ^a^	136/197 69% ^b^	141/210 67% ^b^
	Long agonist, n (%)	33/208 16% ^a^	28/202 14% ^a^	61/197 31% ^b^	69/210 33% ^b^
**Retrieved oocytes** **(mean ± SD, range)**		2.3 ± 1 ^a^	2.5 ± 1.5 ^a^	8.7 ± 3 ^c^	8.4 ± 4 ^c^
**Oocytes MII** **(mean ± SD, range)**		2.01 ± 1 ^a^	2.2 ± 1 ^a^	8.1 ± 2 ^c^	7.8 ± 1 ^c^
**Fertilisation rate** **(%, mean, ± SD)**		54% ^a^ 1.04 ± 0.4	71 ^b^ 1.67 ± 0.3	60% ^a^ 5.3 ± 2	85 ^c^ 6.6 ± 2
**Cleavage rate** **(%, mean, ± SD)**		90% 0.9 ± 0.2	95% 1.6 ± 0.4	98% 5.2 ± 2	97% 6.4 ± 2
**Blastocyst rate** **(%, mean, ± SD)**		38% 0.3+0.1	42% 0.6 ± 2	43% 2.3 ± 0.9	45% 3.5 ± 1
**Cycle with PGT-A** **n (%)**		52/208 25% ^a^	59/202 29% ^a^	86/197 43% ^b^	104/210 49% ^b^
**Euploidy rate (%)**		51%	52%	49%	52%
**Cycle cancel rate** **without ET (%)**		30% ^a^	24% ^a^	9% ^c^	5% ^c^
**Biochemical pregnancy, (%)**		24/135 17% ^a^	70/159 44% ^c^	79/171 46% ^c^	115 59% ^d^
**Clinical pregnancy, n (%)**		22/135 16% ^a^	66/159 41% ^c^	75/171 43% ^c^	110/194 56% ^d^

## Data Availability

The original contributions presented in this study are included in the article. Further inquiries can be directed to the corresponding author.
